# Regular approximate factorization of a class of matrix-function with an unstable set of partial indices

**DOI:** 10.1098/rspa.2017.0279

**Published:** 2018-01-17

**Authors:** G. Mishuris, S. Rogosin

**Affiliations:** 1 Department of Mathematics, Aberystwyth University, Penglais, SY23 3BZ Aberystwyth, UK; 2 Department of Economics, Belarusian State University, Nezavisimosti Avenue, 4, 220030 Minsk, Belarus

**Keywords:** factorization of matrix-functions, asymptotic methods, unstable partial indices

## Abstract

From the classic work of Gohberg & Krein (1958 *Uspekhi Mat. Nauk.* **XIII**, 3–72. (Russian).), it is well known that the set of partial indices of a non-singular matrix function may change depending on the properties of the original matrix. More precisely, it was shown that if the difference between the largest and the smallest partial indices is larger than unity then, in any neighbourhood of the original matrix function, there exists another matrix function possessing a different set of partial indices. As a result, the factorization of matrix functions, being an extremely difficult process itself even in the case of the canonical factorization, remains unresolvable or even questionable in the case of a non-stable set of partial indices. Such a situation, in turn, has became an unavoidable obstacle to the application of the factorization technique. This paper sets out to answer a less ambitious question than that of effective factorizing matrix functions with non-stable sets of partial indices, and instead focuses on determining the conditions which, when having known factorization of the limiting matrix function, allow to construct another family of matrix functions with the same origin that preserves the non-stable partial indices and is close to the original set of the matrix functions.

## 1. Introduction

A given invertible square matrix $G\in (C{\left(R)\right)}^{n\times n}$ is called factorizable if it can be represented in the form

1.1 $$G(x)=G^{-}(x)\Lambda (x)G^{+}(x),$$

with continuous invertible factors *G*^±^(*x*),(*G*^±^)^−1^(*x*), possessing analytic continuation into the corresponding half-plane *Π*^±^={*z*=*x*+*iy*:*Im* ±*z*<0}, and

1.2 $$\Lambda (x)=\mathrm{diag}\;\left({\left(\frac{x-i}{x+i}\right)}^{\varkappa_{1}},\ldots ,{\left(\frac{x-i}{x+i}\right)}^{\varkappa_{n}}\right),\;\varkappa_{1},\ldots ,\varkappa_{n}\in Z.$$

The representation (1.1) is called the *right* (*or right-sided*) *factorization* on the real axis. It is also referred to as right continuous factorization. If we have the representation

1.3 $$G(x)=G^{+}(x)\Lambda (x)G^{-}(x),$$

then it is called the *left* (*or left-sided*) *factorization*. If the right- (or left-) factorization exists, then the integers $\varkappa_{1},\ldots ,\varkappa_{n}$, called the *partial indices*, are determined uniquely up to their order, e.g. $\varkappa_{1}\geq \cdots \geq \varkappa_{n}$. The factors *G*^−^, *G*^+^ are not unique (e.g. [1]). In general, the partial indices for the right-factorization and for the left-factorization are not necessarily the same. Throughout this paper, we will only deal with the right factorization. The case of the left factorization can be handled analogously.

Several of the general facts on factorization have been presented in the survey paper [2] (see also [3,4]). In particular, it is well known that the sum of the partial indices is equal to the winding number (or the Cauchy index) of the determinant of the given invertible square matrix *G*:

1.4 $$\sum_{j=1}^{n}\varkappa_{j}=\varkappa_{G}={\mathrm{wind}}_{R}\;\det\;G(x)=\frac{1}{2\pi }\int_{-\infty }^{+\infty }d(\arg\;\det\;G(x)).$$

The factorization is called a *canonical factorization* if all the partial indices are equal to 0, i.e. $\varkappa_{1}=\cdots =\varkappa_{n}=0$.

It is said (e.g. [5], p. 50) that a non-singular matrix function *G*(*x*) has a *stable set of partial indices* if there exists *δ*>0 such that any matrix function *F*(*x*) from the *δ*-neighbourhood of *G*(*x*) (i.e. ∥*F*−*G*∥<*δ*) has the same set of partial indices (right or left). If not, then *G*(*x*) has an *unstable set of partial indices*. It has been shown (see [5–7] and also [8]) that a set of partial indices $\varkappa_{1},\ldots ,\varkappa_{n}$ is stable if and only if $\varkappa_{1}-\varkappa_{n}\leq 1$.

In the unstable case, a small deformation of the matrix function *G*(*x*) *can* lead to changes in the partial indices. More precisely, there exists a sequence of matrix functions *F*_*k*_(*x*) that converge to *G*(*x*), but which has a distinct set of partial indices, i.e.

1.5 $$F_{k}(x)=F_{k}^{-}(x)\Lambda_{A}(x)F_{k}^{+}(x),$$

where *Λ*_*A*_(*x*)≠*Λ*(*x*).

We note that, in all the known examples illustrating such a situation (e.g. [5,6]), the sequences of the factors $F_{k}^{-}(x)$, $F_{k}^{+}(x)$ *do not* possess limiting values (as k→∞) from the same chosen space as *G*(*x*). On the contrary, even in the case of unstable partial indices, we can easily construct a sequence of factors $F_{k}^{-}(x)$, $F_{k}^{+}(x)$ in (1.5) with the same partial indices as the original matrix, i.e. *Λ*_*A*_(*x*)=*Λ*(*x*). Indeed, in a simple example we present the pair $F_{k}^{-}(x)=G^{-}(x)+\varepsilon_{k}H_{k}^{-}(x)$, $F_{k}^{+}(x)=G^{+}(x)+\varepsilon_{k}H_{k}^{+}(x)$, where $H_{k}^{\mp }$ are arbitrary matrices belonging to the same space as *G*^∓^, such that $∥H_{k}^{\mp }∥\leq h_{0}$ and $\varepsilon_{k}\to 0$ as k→∞.

Let us now consider a family of matrix functions $G\in {GH}_{\mu }{\left(R\right)}^{n\times n}$ that possesses a factorization. Any matrix $G_{\varepsilon }\in {GH}_{\mu }{\left(R\right)}^{n\times n}$ that satisfies the following asymptotic relation:

$$∥G_{\varepsilon }(x)-G(x)∥=O(\varepsilon ),\;\varepsilon \to 0,$$

will be called a *perturbation* of the matrix *G*.

**Definition 1.1**

Let $G\in {GH}_{\mu }{\left(R\right)}^{n\times n}$ be a given factorizable matrix. Its perturbation *G*_*ε*_ is considered ‘regular’, if there exists *ε*_0_>0 such that the matrix *G*_*ε*_ possesses a bounded factorization (i.e. $|G_{\varepsilon }^{\pm }(z)|\leq M$ for all *ε*∈[0,*ε*_0_) and *z*∈*Π*^±^). Otherwise the perturbation is considered ‘singular’.

**Lemma 1.2**

The partial indices of the regular perturbation *G*_*ε*_ are the same as those of *G*.

**Proof.**

Let

$$G(x)=G^{-}(x)\Lambda (x)G^{+}(x),$$

and *G*_*ε*_ be a regular perturbation of *G*(*x*),

$$G_{\varepsilon }(x)=G_{\varepsilon }^{-}(x)\Lambda_{A}(x)G_{\varepsilon }^{+}(x)=G^{-}(x)\Lambda (x)G^{+}(x)+O(\varepsilon ).$$

Hence

$$\Lambda (x)=(G^{-}{\left(x)\right)}^{-1}G_{\varepsilon }^{-}(x)\Lambda_{A}(x)G_{\varepsilon }^{+}(x)(G^{+}{\left(x)\right)}^{-1}+O(\varepsilon ).$$

By taking the limit as *ε*→+0, we obtain *Λ*_*A*_=*Λ* due to the uniqueness of the partial indices. ▪

**Remark 1.3**

If the partial indices of *G* satisfy the condition $\varkappa_{\max}-\varkappa_{\min}\leq 1$, then any perturbation of *G* is regular.

**Remark 1.4**

To highlight the role of the condition of boundedness of the factors, we present here a variant of the classical example of Gohberg & Krein [5], p. 264. Let us consider the following matrix:

1.6 $$G_{0}(x)=\left(\begin{array}{cc} \frac{x-i}{x+i} & 0\\ 0 & \frac{x+i}{x-i} \end{array}\right).$$

It is clear that this matrix possesses a factorization, where $G_{0}^{\pm }(x)=I$, *Λ*(*x*)=*G*_0_(*x*) and the partial indices $\varkappa_{1}=1,\varkappa_{2}=-1$. Consider a slight perturbation of the matrix *G*_0_(*x*):

1.7 $$G_{\varepsilon }(x)=\left(\begin{array}{cc} \frac{x-i}{x+i} & \varepsilon \\ 0 & \frac{x+i}{x-i} \end{array}\right),\;\varepsilon >0.$$

We note that for sufficiently small *ε*>0, the matrices are close to each other, such that

$$∥G_{0}(x)-G_{\varepsilon }(x)∥\leq \varepsilon .$$

On the other hand, for all fixed *ε*>0, the matrix *G*_*ε*_(*x*) possesses the following factorization with partial indices $\varkappa_{1}=\varkappa_{2}=0$:

1.8 $$G_{\varepsilon }(x)=\left(\begin{array}{cc} 1 & 0\\ \frac{1}{\varepsilon }\frac{x+i}{x-i} & 1 \end{array}\right)\cdot I\cdot \left(\begin{array}{cc} \frac{x-i}{x+i} & \varepsilon \\ -\frac{1}{\varepsilon } & 0 \end{array}\right).$$

For each fixed *ε*>0, the factors in this factorization admit analytic continuations into the corresponding half-plane, where they are bounded. However, these factors are not uniformly bounded for *ε*∈[0,*ε*_0_) for any *ε*_0_>0. Hence *G*_*ε*_(*x*) is a singular perturbation of the above diagonal matrix *G*_0_(*x*) (we denote it by $G_{\varepsilon }^{\left(s\right)}(x)$).

**Figure 1.**
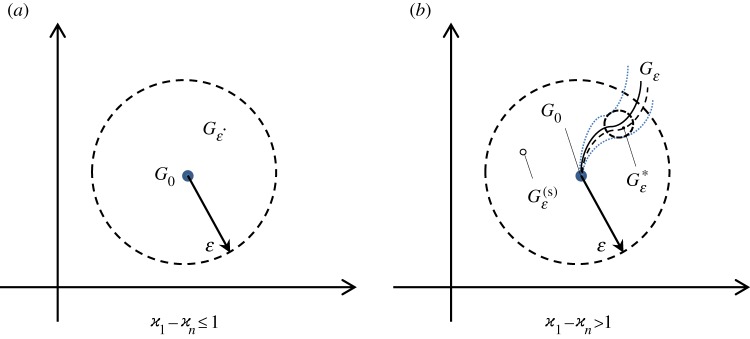
Possible types of perturbations, *G*_*ε*_, (∥*G*_*ε*_−*G*_0_∥<*ε*), in the cases of stable (*a*) and unstable (*b*) sets of partial indices of the matrix-function *G*_0_. (Online version in colour.)

This example provides a simple, but not unique, method for the construction of the singular perturbation $G_{\varepsilon }^{\left(s\right)}(x)$ of any *n*×*n* diagonal matrix $\Lambda (x)=\mathrm{diag}\{((x-i)/{\left(x+i)\right)}^{\varkappa_{1}},\ldots ,((x-i)/{\left(x+i)\right)}^{\varkappa_{n}}\}$. Moreover, by replacing *ε* with *ε*^*k*^ in this procedure, we can construct a singular perturbation for any factorizable matrix $G_{0}(x)=G_{0}^{-}(x)\Lambda (x)G_{0}^{+}(x)$ (figure 1),

1.9 $$G_{\varepsilon }^{\left(s\right)}(x)=G_{0}^{-}(x)\Lambda_{\varepsilon }^{\left(s\right)}(x)G_{0}^{+}(x),$$

which is arbitrarily close to the given matrix *G*_0_(*x*).

**Definition 1.5**

Let *G*_*ε*_(*x*) be a perturbation of a factorizable matrix function *G*_0_(*x*). If there exists another perturbation $G_{\varepsilon }^{*}(x)$ satisfying

$$∥G_{\varepsilon }(x)-G_{\varepsilon }^{*}(x)∥=O(\varepsilon^{k}),\;\varepsilon \to 0,$$

then we say that $G_{\varepsilon }^{*}$ is a ‘*k*-guided perturbation’ of *G*_*ε*_.

It follows from (1.9) that for each regular perturbation *G*_*ε*_(*x*) of the matrix function *G*_0_(*x*), there exists a singular *k*-guided perturbation $G_{\varepsilon }^{\left(s\right)}(x)$ for any *k*≥1.

The above-mentioned properties are illustrated in figure 1. In figure 1*a*, we show that for any factorizable matrix *G*_0_(*x*), with a stable set of partial indices, there is a *ε*-neighbourhood containing only regular perturbations. Figure 1*b* illustrates the case of unstable partial indices of *G*_0_(*x*). Here, the situation is more delicate, as we can see that in each *ε*-neighbourhood of *G*_0_(*x*) we can find either regular or singular perturbations.

The aim of this paper is to consider the construction of a regular *k*-guided (*k*>1) perturbation $G_{\varepsilon }^{*}(x)$ for a given perturbation *G*_*ε*_(*x*) of the matrix function *G*_0_(*x*). Perturbation *G*_*ε*_(*x*) is of the same type as in [9,10], and *G*_0_(*x*) has a known factorization with unstable partial indices. For *k*=1, this is trivial but has no practical use.

The notion of *k*-guided perturbation helps us to highlight possible structure of the set of partial indices of those matrices locating in *ε*-neighbourhood of a matrix with unstable partial indices. Clearly, with the higher *k*-guided perturbation is possible to construct the more accurate approximation of the original set it creates.

The factorization technique is a powerful tool in solving practical problems [11–18], and any approximate factorization will allow a wide range of practical problems to be tackled with some level of accuracy. The establishment of an approximate (e.g. [19]) or an asymptotic procedure [9,10,20–22] is a challenging problem, because an exact factorization is possible only in a number of special cases (see [2] and references therein). Similarly, the mentioned non-uniqueness of the factorization problem does not prevent it being effectively used in practice. However, one needs to be careful in using the approximate factorization in the case of unstable indices, as it may introduce not only quantitative but also qualitative deviation of the approximate solution from the original one. Here, any links between the partial indices of the factorization problem and the particular physical properties of the problem in question are crucial (see extended discussion in [2], cf. also [25,26]). This question is beyond the scope of this paper.

Below, we discuss whether, and under which conditions, it is possible to find an *n*×*n* matrix function $G_{\varepsilon }^{*}(x),x\in R$, sufficiently close to a given regular perturbation *G*_*ε*_(*x*) of the matrix function *G*_0_(*x*), and possessing an unstable set of partial indices. More exactly, we ask when it is possible to find $G_{\varepsilon }^{*}(x)$ while preserving the partial indices of *G*_*ε*_(*x*)? To reach an answer to this question in the case of unstable partial indices, a new definition of the asymptotic factorization is given and applied. The method, as proposed in [9,10], is generalized and employed. We find conditions under which our asymptotic procedure is effective, and its properties and details are illustrated by examples. The efficiency of the procedure is also illuminated by numerical results.

The paper is organized as follows: In §2, we present necessary definitions and notations supplied by necessary basic facts from factorization theory. Next, (§3) we consider certain perturbations of matrices factorized with unstable partial indices, and describe an algorithm for the construction of an approximate factorization of the perturbed matrices, while preserving the initial partial indices. The conditions for realization of this algorithm are also derived here, which are simply *solvability conditions* for a certain boundary value problem. We also provide examples where the solvability conditions are satisfied and unsatisfied. We conclude with illustrations of the obtained numerical results and a discussion of their importance in §4.

## 2. Asymptotic factorization: definitions

To proceed, we make some necessary definitions. We denote by $H_{\mu }{\left(R\right)}^{n\times n}$, n∈N, the set of bounded matrix functions with locally Hölder-continuous entries, endowed with the norm ∥⋅∥_*μ*_:

2.1 $$H_{\mu }{\left(R\right)}^{n\times n}=\left\{G=(g_{ij}):R\to M^{n\times n}:∥G{∥}_{\mu }=\max_{1\leq i,j\leq n}∥g_{ij}{∥}_{\mu }<\infty \right\}.$$

In this paper, we consider only matrices of the class $GH_{\mu }{\left(R\right)}^{n\times n}$, where G refers to *invertible* matrices. It should be noted that our method can be also be applied to a wider class of matrix functions, e.g. having no prescribed modulus of continuity.

Below, we give a definition of the asymptotic factorization only in the case of the regular perturbation of a given matrix function, since we lack for the moment a formal procedure which distinguishes between the cases of regular and singular perturbations and as yet have no useful example of the construction of the asymptotic factorization of a singularly perturbed matrix function.

**Definition 2.1**

Let $G_{0}(x)\in GH_{\mu }{\left(R\right)}^{n\times n}$ be a given factorizable matrix ($G_{0}(x)=G_{0}^{-}(x)\Lambda (x)G_{0}^{+}(x)$) and *G*_*ε*_(*x*) be its regular perturbation. We say that a set of pairs of matrix functions, $G_{\varepsilon ,m}^{-}(x)$ and $G_{\varepsilon ,m}^{+}(x)$, (*m*=1,2,…*N*), and a diagonal matrix *Λ*_*A*_(*x*) of the form (1.2) represent an asymptotic factorization (of the order N) of the matrix function $G(x)\in GH_{\mu }{\left(R\right)}^{n\times n}$ if the following conditions are satisfied:

(i) there exists a sequence of functions *θ*_*k*_(*ε*), *k*=1,2,…*N*+1, that vanishes at the point *ε*=0, such that for any *k*=1,2,…,*N*
  2.2 $$\theta_{k+1}(\varepsilon )=o(\theta_{k}(\varepsilon )),\;\varepsilon \to 0;$$
(ii) there exist matrices $G_{\varepsilon ,m}^{\mp }(x)$ of the form:
  2.3 $$G_{\varepsilon ,m}^{\mp }(x)={G_{0}}^{\mp }(x)+\sum_{k=1}^{m}\theta_{k}(\varepsilon )H_{\varepsilon ,k}^{\mp }(x),$$
(iii) there exists *ε*_0_>0 such that the matrices $H_{\varepsilon ,k}^{\mp }(z)$ are analytical in *Π*^∓^, respectively, and bounded in $H_{\mu }{\left(R\right)}^{n\times n}$ uniformly with respect to *ε*∈[0,*ε*_0_),
(iv) the following estimate is valid for any *m*=1,2,…,*N*
  2.4 $$G_{0}^{-}(x)\Lambda (x)G_{0}^{+}(x)-G_{\varepsilon ,m}^{-}(x)\Lambda_{A}(x)G_{\varepsilon ,m}^{+}(x)=O(\theta_{m+1}(\varepsilon )),\;\varepsilon \to 0.$$

The representation

2.5 $$G_{\varepsilon }^{*}(x)=G_{\varepsilon ,N}^{-}(x)\Lambda_{A}(x)G_{\varepsilon ,N}^{+}(x)$$

is called an asymptotic factorization (of order *N*) of the matrix *G*_*ε*_(*x*).

We note that conditions (2.2) and (2.3) guarantee that the matrices $G_{\varepsilon ,m}^{\mp }(z)$ and $(G_{\varepsilon ,m}^{\mp }{\left(z)\right)}^{-1}$ belong to the required class, and thus both terms on the left-hand side of (2.4) represent two essentially different factorizations. As a simple example of the sequence (2.2), we could consider *θ*_*k*_(*ε*)=*ε*^*k*^.

Some clarifications are required for this definition:

— We are concerned with *regular* perturbations of the given matrix, i.e. we are looking for representations (2.3) possessing factors $G_{\varepsilon ,m}^{-}(z)$ and $G_{\varepsilon ,m}^{+}(z)$ that are bounded in *z* in the corresponding half-planes, where our choice is motivated purely by applications. In fact, we can replace the boundedness conditions for $G_{\varepsilon ,m}^{-}(z)$ and $G_{\varepsilon ,m}^{+}(z)$, by other conditions, such as polynomial growth/decay at infinity.
— The given definition does not require uniqueness. Indeed, as was demonstrated in the example in [10], which considers the case of stable partial indices, there was no uniqueness, even with the enforcement of additional conditions at infinity.
— The parameter *N* is also involved in the process of asymptotic factorization. In the case when N=∞ and the series is converging, we can say that the asymptotic factorization becomes the standard factorization, where the factors are defined by their converging series.
— The method used in [10], in the case of stable partial indices, allows to construct for some matrix functions the factors of the factorization as converging asymptotic series, and preserving the partial indices, i.e. *Λ*_*A*_(*x*)=*Λ*(*x*). However, even in this case, no uniqueness can be guaranteed.
— The factors $G_{\varepsilon ,m}^{\mp }(x)$ in the representation (2.3) are continuous with respect to *ε*≥0.
— Although the asymptotic factorization is not unique, we can prove, similarly to lemma 1.2, the uniqueness of the partial indices (*Λ*_*A*_(*x*)=*Λ*(*x*)).
— If an asymptotic factorization of order *N*>1 exists, then the matrix function
  $$G_{\varepsilon }^{*}(x)=G_{\varepsilon ,N}^{-}(z)\Lambda (x)G_{\varepsilon ,N}^{+}(z)$$
  is an (*N*+1)-guided perturbation of the following perturbation *G*_*ε*_(*x*) of the matrix *G*(*x*):
  $$G_{\varepsilon }(x)=G_{\varepsilon ,1}^{-}(z)\Lambda (x)G_{\varepsilon ,1}^{+}(z).$$

Although we only consider in this paper the factorization of a matrix function on the real axis, we can tackle in the same way the factorization of matrices defined on any oriented curve *Γ* which divides the complex plane into two domains *D*^−^ and *D*^+^, by changing the diagonal entries in *Λ*(*x*) to $((x-t^{+})/{\left(x-t^{-})\right)}^{\varkappa_{j}}$, *t*^∓^∈*D*^∓^, or simply to $x^{\varkappa_{j}}$ if 0∈*D*^+^.

Let us now consider a matrix function $G_{0}\in GH_{\mu }{\left(R\right)}^{n\times n}$, admitting a factorization (1.1), with unstable partial indices $\varkappa_{1},\ldots ,\varkappa_{n}$ ($\varkappa_{1}\geq \cdots \geq \varkappa_{n}$), and its perturbation $G_{\varepsilon }(x)\in GH_{\mu }{\left(R\right)}^{n\times n}$, which depends on a small parameter *ε* such that

2.6 $$G_{\varepsilon }(x){|}_{\varepsilon =0}=G_{0}(x).$$

Our motivating question is the following: how to distinguish a class of possible perturbations that allows constructing an asymptotic procedure described in (2.2)–(2.5). In the case of stable partial indices, such a type of perturbation and the corresponding asymptotic procedure was always possible as shown in [9,10]. We demonstrate below that the statement is no longer valid in the case of unstable partial indices and some additional conditions are required.

## 3. Asymptotic factorization: procedure

Let us consider an invertible, bounded, locally Hölder continuous matrix $G_{\varepsilon }(x):R\to {GH}_{\mu }{\left(R\right)}^{n\times n}$ of the form

3.1 $$G_{\varepsilon }(x)=G_{0}(x)+\theta_{1}(\varepsilon )N_{\varepsilon }(x),$$

where *θ*_1_(*ε*)=*o*(1) as *ε*→0 and *N*_*ε*_ is bounded and Hölder continuous on R. We suppose additionally that, when *ε*=0, the matrix *G*_0_(*x*) possesses a factorization with unstable partial indices ($\varkappa_{1}-\varkappa_{n}\geq 2$), and has factors $G_{0}^{\mp }(x)$ and $(G_{0}^{\mp }{\left(x)\right)}^{\pm 1}$, which admit analytic continuation into the semi-planes *Π*^∓^, respectively, and which are bounded in ${\bar{\Pi }}^{\mp }=\Pi^{\mp }\cup R$.

We look for an asymptotic factorization of the matrix *G*_*ε*_(*x*) of the type (3.1), which is a regular perturbation of *G*_0_(*x*), up to some stage of the asymptotic procedure. For simplicity, we will consider *θ*_*k*_(*ε*)=*ε*^*k*^ (see remark 3.6, cf. [9], Lemma 3.6).

### (a) First step of the asymptotic factorization

First, we present the matrix *G*_*ε*_(*x*) in the following form:

3.2 $$\begin{array}{rl} G_{\varepsilon }(x) & =(G_{0}^{-}(x)+\varepsilon N_{1,\varepsilon }^{-}(x)(\Lambda^{+}{\left(x)\right)}^{-1})\Lambda (x)\\ & \;\times (G_{0}^{+}(x)+\varepsilon (\Lambda^{-}{\left(x)\right)}^{-1}N_{1,\varepsilon }^{+}(x))+O(\varepsilon^{2}), \end{array}$$

where $\Lambda^{\mp }(x)=\mathrm{diag}(((x-i)/{\left(x+i)\right)}^{\varkappa_{1}^{\mp }},\ldots ,((x-i)/{\left(x+i)\right)}^{\varkappa_{n}^{\mp }})$, $\varkappa_{j}^{+}=\max\{\varkappa_{j},0\},\varkappa_{j}^{-}=\max\{0,-\varkappa_{j}\}$, and the unknown matrix-functions $N_{1,\varepsilon }^{\mp }(x)$ must be analytically extended into *Π*^∓^, together with their inverses, and bounded in ${\bar{\Pi }}^{\mp }$, respectively. Note that (3.2) differs from the representation used for the case of stable partial indices (cf. [9,10]).

Comparing the term with parameter *ε*, we arrive at the following boundary condition for $N_{1,\varepsilon }^{\mp }$:

3.3 $$N_{1,\varepsilon }^{-}(x)\Lambda^{-}(x)G_{0}^{+}(x)+G_{0}^{-}(x)\Lambda^{+}(x)N_{1,\varepsilon }^{+}(x)=N_{\varepsilon }(x).$$

For brevity, we introduce the following notation:

3.4 $$\begin{array}{rl} \tilde{N_{1,\varepsilon }^{-}}(x) & :=({\tilde{n}}_{1,ij}^{-}{\left(z)\right)}_{ij}=(G_{0}^{-}{\left(x)\right)}^{-1}N_{1,\varepsilon }^{-}(x),\\ \tilde{N_{1,\varepsilon }^{+}}(x) & :=({\tilde{n}}_{1,ij}^{+}{\left(z)\right)}_{ij}=N_{1,\varepsilon }^{+}(x)(G_{0}^{+}{\left(x)\right)}^{-1}\\ \;\text{and}\;M_{0,\varepsilon }(x) & :=(m_{0,ij}{\left(z)\right)}_{ij}=(G_{0}^{-}{\left(x)\right)}^{-1}N_{\varepsilon }(x)(G_{0}^{+}{\left(x)\right)}^{-1}. \end{array}\}$$

Hence, unknown matrix-functions $\tilde{N_{1,\varepsilon }^{\mp }}$ have to satisfy the boundary condition

3.5 $$\tilde{N_{1,\varepsilon }^{-}}(x)\Lambda^{-}(x)+\Lambda^{+}(x)\tilde{N_{1,\varepsilon }^{+}}(x)=M_{0,\varepsilon }(x),\;x\in R.$$

In order to determine solvability conditions for this problem and to find a representation for the solution, we present here a few facts from the theory of boundary value problems [23,24]. It is known that any bounded locally Hölder continuous function f:R→C can be uniquely, up to arbitrary constant c∈C, represented as the sum of two functions which are analytic in *Π*^−^ and *Π*^+^, an bounded in ${\bar{\Pi }}^{-}$ and ${\bar{\Pi }}^{+}$, respectively,

3.6 $$f(x)=((\Omega_{0}^{-}f)(x)+c)+((\Omega_{0}^{+}f)(x)-c),$$

where $\left(\Omega_{0}^{\mp }f)(z\right)$ is the Cauchy-type integral [23], p. 52

3.7 $$(\Omega_{0}^{\mp }f)(z)=\mp \frac{z-i}{2\pi i}\int_{-\infty }^{+\infty }\frac{f(\tau )\;d\tau }{\left(\tau -i)(\tau -z\right)},\;z\in \Pi^{\mp }.$$

Representation (3.6), and further formulae, remain valid in the matrix case too. Therefore, the *formal* solution to (3.5) has the following form:

3.8 $$\tilde{N_{1,\varepsilon }^{-}}(z)=[(\Omega_{0}^{-}M_{0,\varepsilon })(z)+C^{0}](\Lambda^{-}{\left(z)\right)}^{-1}$$

and

3.9 $$\tilde{N_{1,\varepsilon }^{+}}(z)=(\Lambda^{+}{\left(z)\right)}^{-1}[(\Omega_{0}^{+}M_{0,\varepsilon })(z)-C^{0}],$$

where *C*^0^ is a constant *n*×*n* matrix, which is, in coordinate-wise terms,

3.10 $$\begin{array}{rl} {\tilde{n}}_{1,lj}^{-}(z) & =(\Omega_{0}^{-}m_{0,lj})(z)+c_{lj}^{0},\;1\leq l\leq n,1\leq j\leq q\\ \;\mathrm{and}\;{\tilde{n}}_{1,lj}^{-}(z) & ={\left(\frac{z-i}{z+i}\right)}^{-\varkappa_{j}}[(\Omega_{0}^{-}m_{0,lj})(z)+c_{lj}^{0}],\;1\leq l\leq n,q+1\leq j\leq n; \end{array}\}$$

and

3.11 $$\begin{array}{rl} {\tilde{n}}_{1,lj}^{+}(z) & ={\left(\frac{z+i}{z-i}\right)}^{\varkappa_{l}}[(\Omega_{0}^{+}m_{0,lj})(z)-c_{lj}^{0}],\;1\leq l\leq p,1\leq j\leq n\\ \;\mathrm{and}\;{\tilde{n}}_{1,lj}^{+}(z) & =(\Omega_{0}^{+}m_{0,lj})(z)-c_{lj}^{0},\;p+1\leq l\leq n,1\leq j\leq n. \end{array}\}$$

Such a representation gives the bounded solution of the first-order asymptotic factorization problem (3.2), with factors involving analytic matrices $N_{1,\varepsilon }^{\mp }$ which are uniquely defined by (3.4), if and only if certain solvability conditions are satisfied. These conditions simply require that $\tilde{N_{1,\varepsilon }^{\mp }}(z)$ have no singular points at ∓*i*. Partly, we can use arbitrary constant $c_{lj}^{0}$ (the entries of the matrix *C*^0^), but not all the solvability conditions are satisfied by the proper choice of $c_{lj}^{0}$.

### (b) Solvability conditions

Here, we present the necessary and sufficient solvability conditions for boundary value problem (3.5), which is equivalent to the first step of the asymptotic factorization [23], p. 120.

— if for certain *k*,*q*+1≤*k*≤*n*, we have $\varkappa_{k}=-1$, then the boundedness of ${\tilde{N}}_{1,\varepsilon }^{-}(z)$ at *z*=−*i* follows whenever we choose $c_{lk}^{0}$ such that
  3.12 $$c_{lk}^{0}=\frac{1}{\pi }\int_{-\infty }^{+\infty }\frac{m_{0,lk}(\tau )\;d\tau }{\tau^{2}+1},\;1\leq l\leq n;$$
— if for certain *k*,*q*+1≤*k*≤*n*, we have $\varkappa_{k}<-1$, then the corresponding $c_{lk}^{0}$ must be chosen as in (3.12), and the entries *m*_0,*lk*_(*τ*) have to satisfy conditions
  3.13 $$\int_{-\infty }^{+\infty }\frac{m_{0,lk}(\tau )\;d\tau }{{\left(\tau +i\right)}^{r+1}}=0,\;1\leq r\leq -\varkappa_{k}-1,\;1\leq l\leq n;$$
— if for certain *k*,1≤*k*≤*p*, we have $\varkappa_{k}=1$, then the boundedness of $\tilde{N_{1,\varepsilon }^{+}}(z)$ at *z*=*i* follows whenever we choose $c_{kj}^{0}$ such that
  3.14 $$c_{kj}^{0}=0,\;1\leq j\leq n;$$
— if for certain *k*,1≤*k*≤*p*, we have $\varkappa_{k}>1$, then the corresponding $c_{kj}^{0}$ must be chosen as in (3.14), and the entries *m*_0,*kj*_(*τ*) have to satisfy conditions
  3.15 $$\int_{-\infty }^{+\infty }\frac{m_{0,kj}(\tau )\;d\tau }{{\left(\tau -i\right)}^{r+1}}=0,\;1\leq r\leq \varkappa_{k}-1,\;1\leq j\leq n;$$
— if the pair (*l*,*j*) is such that 1≤*l*≤*p*,*q*+1≤*j*≤*n*, then additional solvability conditions must satisfy
  3.16 $$\int_{-\infty }^{+\infty }\frac{m_{0,lj}(\tau )\;d\tau }{\tau^{2}+1}=0,\;1\leq l\leq p,q+1\leq j\leq n;$$
— if the pair (*l*,*j*) is such that either 1≤*l*≤*n*,1≤*j*≤*q*, or *p*+1≤*l*≤*n*,1≤*j*≤*n*, then we have no condition on the entries *m*_0,*lj*_(*τ*); the corresponding constants $c_{lj}^{0}$ can take arbitrary value.

**Theorem 3.1**

*Formula (*3.2*) gives the first-order bounded asymptotic factorization for all ε smaller than a certain positive ε*_1_ *if and only if the solvability conditions (*3.13*), (*3.15*) and (*3.16*) are satisfied, and the constants* $c_{ij}^{0}$ *are chosen accordingly.*

**Proof.**

If the conditions of the theorem are satisfied, then the matrix functions $\tilde{N_{1,\varepsilon }^{\mp }}(z)$ give a bounded solution to the problem (3.5). Moreover, the matrices $\tilde{N_{1,\varepsilon }^{-}}(z)\Lambda^{-}(z)$, $\Lambda^{+}(z)\tilde{N_{1,\varepsilon }^{+}}(z)$ are bounded in the neighbourhoods of *z*=−*i*, *z*=*i*, respectively. By choosing sufficiently small *ε*_1_>0, we can guarantee that the matrix functions $G_{0}^{-}(z)+\varepsilon N_{1,\varepsilon }^{-}(z)(\Lambda^{+}{\left(z)\right)}^{-1}$, $G_{0}^{+}(z)+\varepsilon (\Lambda^{-}{\left(z)\right)}^{-1}N_{1,\varepsilon }^{+}(z)$ are invertible in the corresponding semi-planes. Thus, for *ε*∈[0,*ε*_1_), formula (3.2) gives the first-order bounded asymptotic factorization.

To demonstrate the necessity of the theorem’s conditions, we suppose that formula (3.2) gives the first-order bounded asymptotic factorization. Then the matrix functions $N_{1,\varepsilon }^{\mp }(z)$ have to satisfy boundary condition (3.3), being analytically extended into *Π*^∓^ together with their inverses, and bounded in ${\bar{\Pi }}^{\mp }$, respectively. The boundary value problem (3.3) is equivalent to (3.5), and for invertibility of matrices $N_{1,\varepsilon }^{\mp }(z)$ we must, in particular, have boundedness of the matrix functions (3.8) and (3.9) in the neighbourhoods of *z*=−*i* and *z*=*i*, respectively. The latter leads to the necessity of the conditions of the theorem. ▪

**Remark 3.2**

The numbers of solvability conditions and conditions on the choice of the constants satisfy the following relations.

— The number of solvability conditions is given by
  3.17 $$\sum_{j=q+1}^{n}(-\varkappa_{j}-1)n+\sum_{i=1}^{p}(\varkappa_{i}-1)n+(n-q)p.$$
— (*n*−*q*)*n* constants $c_{ij}^{0}$ are chosen according to (3.12) and *np* constants $c_{ij}^{0}$ are equal to 0, as in (3.14). In the (*n*−*q*)*p* cases described in (3.16) these choices of the constants $c_{ij}^{0}$ must coincide.
— *n*(*n*−*p*+*q*) constants $c_{ij}^{0}$ can be chosen arbitrarily.

**Remark 3.3**

The obtained result can be interpreted in the following manner. Let the matrix function *G*_*ε*_(*x*) be a perturbation of *G*_0_(*x*). Then, in particular, *G*_*ε*_(*x*) is in the *ε*-neighbourhood of *G*_0_(*x*) (figure 1*a*). If the matrix *G*_*ε*_(*x*) satisfies the above solvability conditions, then there exists for all sufficiently small *ε* the matrix

3.18 $$G_{\varepsilon }^{*}(x)=(G_{0}^{-}(x)+\varepsilon N_{1,\varepsilon }^{-}(x)(\Lambda^{+}{\left(x)\right)}^{-1})\Lambda (x)(G_{0}^{+}(x)+\varepsilon (\Lambda^{-}{\left(x)\right)}^{-1}N_{1,\varepsilon }^{+}(x)),$$

which possesses a factorization with the same unstable set of partial indices as *G*_0_(*x*). The matrix $G_{\varepsilon }^{*}(x)$ is in the *ε*^2^-neighbourhood of *G*_*ε*_(*x*) (figure 1*b*). This means that for each point of linear manifold of the matrices *G*_*ε*_(*x*), as defined by (3.2), which satisfies the solvability conditions, there exists a point (matrix $G_{\varepsilon }^{*}(x)$) in its *ε*^2^-neighbourhood which preserves the initial partial indices, i.e. according to definition 1.5, the latter matrix is the regular 2-guided perturbation.

### (c) Further steps of the asymptotic factorization

Let the solvability conditions be satisfied and the constants $c_{ij}^{0}$ chosen accordingly. By solving the corresponding boundary value problems, we can refine the first-order factorization up to the *r*th step of the factorization using the representation

3.19 $$\begin{array}{rl} G_{\varepsilon }(x) & =(G_{0}^{-}(x)+\varepsilon N_{1,\varepsilon }^{-}(x)(\Lambda^{+}{\left(x)\right)}^{-1}+\cdots +\varepsilon^{r}N_{r,\varepsilon }^{-}(x)(\Lambda^{+}{\left(x)\right)}^{-1})\Lambda (x)\\ & \;\times (G_{0}^{+}(x)+\varepsilon (\Lambda^{-}{\left(x)\right)}^{-1}N_{1,\varepsilon }^{+}(x)+\ldots +\varepsilon^{r}(\Lambda^{-}{\left(x)\right)}^{-1}N_{r,\varepsilon }^{+}(x))+O(\varepsilon^{r+1}), \end{array}$$

which leads to the boundary value problem

3.20 $$\tilde{N_{r,\varepsilon }^{-}}(x)\Lambda^{-}(x)+\Lambda^{+}(x)\tilde{N_{r,\varepsilon }^{+}}(x)=M_{r-1,\varepsilon }(x),\;x\in R$$

and

3.21 $$\tilde{N_{r,\varepsilon }^{-}}(x):=(G_{0}^{-}{\left(x)\right)}^{-1}N_{r,\varepsilon }^{-}(x),\;\tilde{N_{r,\varepsilon }^{+}}(x):=N_{r,\varepsilon }^{+}(x)(G_{0}^{+}{\left(x)\right)}^{-1},$$

$$M_{r-1,\varepsilon }(x):=-(G_{0}^{-}{\left(x)\right)}^{-1}[N_{1,\varepsilon }^{-}(x)N_{r-1,\varepsilon }^{+}(x)+\cdots +N_{r-1,\varepsilon }^{-}(x)N_{1,\varepsilon }^{+}(x)](G_{0}^{+}{\left(x)\right)}^{-1}.$$

The *formal* solution to problem (3.20) can be presented as

3.22 $$\tilde{N_{r,\varepsilon }^{-}}(z)=[(\Omega_{0}^{-}M_{r-1,\varepsilon })(z)+C^{r-1}](\Lambda^{-}{\left(z)\right)}^{-1}$$

and

3.23 $$\tilde{N_{r,\varepsilon }^{+}}(z)=(\Lambda^{+}{\left(z)\right)}^{-1}[(\Omega_{0}^{+}M_{r-1,\varepsilon })(z)-C^{r-1}],$$

which features a new constant matrix *C*^*r*−1^. It becomes the solution for the considered class if and only if the solvability conditions (3.12)–(3.16) are satisfied (in this case, we replace the functions *m*_0,*ij*_(*x*) with the functions *m*_*r*−1,*ij*_(*x*), and the constants $c_{ij}^{0}$ with the constants $c_{ij}^{r-1}$), while the constants $c_{ij}^{r-1}$ are chosen accordingly.

If at a certain step *r*=*N*+1, at least one solvability condition fails, then the procedure for the asymptotic factorization is stopped at this point.

**Remark 3.4**

In this case, we summarize the situation as follows. Let the matrix function *G*_*ε*_(*x*) be a regular perturbation of *G*_0_(*x*). In particular, (as for *N*=1) *G*_*ε*_(*x*) is in the *ε*-neighbourhood of *G*_0_(*x*). If the matrix function *G*_*ε*_(*x*) satisfies the above solvability conditions at each step *r*,1≤*r*≤*N*, then for all sufficiently small *ε* there exists a matrix

3.24 $$G_{N,\varepsilon }^{*}(x)=(G_{0}^{-}(x)+\sum_{r=1}^{N}\varepsilon^{r}N_{r,\varepsilon }^{-}(x)(\Lambda^{+}{\left(x)\right)}^{-1})\Lambda (x)(G_{0}^{+}(x)+\sum_{r=1}^{N}\varepsilon^{r}(\Lambda^{-}{\left(x)\right)}^{-1}N_{r,\varepsilon }^{+}(x)),$$

which possesses a factorization with the same set of unstable partial indices as *G*_0_(*x*). The matrix $G_{N,\varepsilon }^{*}(x)$ is in the *ε*^*N*+1^-neighbourhood of *G*_*ε*_(*x*). This means that for each point of linear manifold of the matrices *G*_*ε*_(*x*) that satisfies the solvability conditions, there exists an (*N*+1)-guided perturbation. Thus, with a larger number of steps we can proceed in our approximate factorization more closely to the index-preserving approximation to a given matrix *G*_*ε*_(*x*).

**Remark 3.5**

If at least one solvability condition fails at the *N*th step of the approximation, then we can only construct an approximate factorization up to the order *N*−1. If a solvability condition fails at the first step of the approximation, then we do not have a tool to construct a regular *k*-guided perturbation for any *k*>1.

### (d) Example of the perturbed matrix satisfying the first-order solvability conditions

We apply the above-described asymptotic procedure to the matrix function *G*_*ε*_(*x*) of the form

3.25 $$G_{\varepsilon }(x)=\left(\begin{array}{cc} \frac{x^{2}+xi(-18+8\;e^{i\varepsilon x}+8\;e^{-i\varepsilon x})-1}{x^{2}+1} & \frac{xi(24-12\;e^{i\varepsilon x}-12\;e^{-i\varepsilon x})}{x^{2}+1}\\ \frac{xi(-12+4\;e^{i\varepsilon x}+8\;e^{-i\varepsilon x})}{x^{2}+1} & \frac{x^{2}+xi(18-8\;e^{i\varepsilon x}-8\;e^{-i\varepsilon x})-1}{x^{2}+1} \end{array}\right)$$

and show that this matrix possesses an asymptotic factorization with the same partial indices as *G*_0_(*x*). Here the matrix function *G*_0_(*x*) is given by (1.6).

The matrix function *G*_*ε*_(*x*) can be represented in the following form:

3.26 $$G_{\varepsilon }(x)=\Lambda (x)+N_{\varepsilon }(x),$$

where *Λ*(*x*) is the same as in remark 1.4 and the matrix function *N*_*ε*_(*x*) is given by

3.27 $$N_{\varepsilon }(x)=\left(\begin{array}{cc} \frac{xi(-16+8\;e^{i\varepsilon x}+8\;e^{-i\varepsilon x})}{x^{2}+1} & \frac{xi(24-12\;e^{i\varepsilon x}-12\;e^{-i\varepsilon x})}{x^{2}+1}\\ \frac{xi(-12+4\;e^{i\varepsilon x}+8\;e^{-i\varepsilon x})}{x^{2}+1} & \frac{xi(16-8\;e^{i\varepsilon x}-8\;e^{-i\varepsilon x})}{x^{2}+1} \end{array}\right)$$

or

Nε(x)=(−32xi sin2⁡(εx/2)x2+148xi sin2⁡(εx/2)x2+1−24xi sin⁡(εx/2)(sin⁡(εx/2)−icos⁡(εx/2))x2+132xi sin2⁡(εx/2)x2+1)=x sin⁡(εx/2)x2+1(−32i sin⁡εx248i sin⁡εx2−24i e−i(εx/2)32i sin⁡εx2).

Thus, *G*_*ε*_(*x*) can be thought of as a small perturbation of the matrix function *G*_0_(*x*)=*Λ*(*x*) ($G_{0}^{\pm }(x)=I$). The matrix function *N*_*ε*_(*x*) takes the following representation (uniform in x∈R and in *ε*) on any finite interval:

Nε(x)=ϕ(x,ε)N~ε(x),ϕ(x,ε)=x sin⁡(εx/2)x2+1,

where ${\tilde{N}}_{\varepsilon }(x)$ is a bounded matrix.

**Remark 3.6**

The introduced small parameter *ϕ*(*x*,*ε*) has the following properties (cf. [9], Lemma 3.6):

3.28 $$\phi (x,\varepsilon )=O(\varepsilon ),\;\forall \;0<\varepsilon <\varepsilon_{0}$$

and

3.29 $$\phi (x,\varepsilon )=O\left(\frac{1}{x}\right),\;|x|\to +\infty .$$

We note here that $\theta_{1}(\varepsilon )=\max_{x\in \bar{R}}\phi (x,\varepsilon )$. In our case, we can prove that *θ*_1_(*ε*)=*O*(*ε*). We can thus later use an artificial small parameter *ε* instead of *θ*_1_(*ε*).

**Remark 3.7**

In fact, the first-order decay of *ϕ*(*x*,*ε*) at infinity (3.29) is crucial to the behaviour of *θ*_1_(*ε*) with respect to *ε*. If, for example, $\phi (x,\varepsilon )=O(1/\sqrt{x})$, then it leads to only *θ*_1_(*ε*)=*O*(*ε*^1/2^).

The first step of the asymptotic factorization procedure.

We look for a pair of matrix functions $N_{1,\varepsilon }^{\pm }(x)$, which forms an approximate solution, up to *ε*^1^, of the functional equation

3.30 $$G_{\varepsilon }(x)=(I+N_{1,\varepsilon }^{-}(x)(\Lambda^{+}{\left(x)\right)}^{\left(-1\right)})\Lambda (x)(I+(\Lambda^{-}{\left(x)\right)}^{\left(-1\right)}N_{1,\varepsilon }^{+}(x))+O(\varepsilon^{2}).$$

We remind here that $G_{0}^{\pm }(x)=I$ (cf. (3.3)). The approximate solution to (3.30) can be found from the matrix boundary value problem (3.5) that takes in this case the form:

3.31 $$\Lambda^{+}(x)N_{1,\varepsilon }^{+}(x)+N_{1,\varepsilon }^{-}(x)\Lambda^{-}(x)=N_{\varepsilon }(x),$$

where *M*_0,*ε*_(*x*)=*N*_*ε*_(*x*).

Bounded solutions to (3.31) have to satisfy the relation

3.32 $$\Lambda^{+}(x)N_{1,\varepsilon }^{+}(x)=M_{0,\varepsilon }^{+}(x)-C_{0},\;\Lambda^{-}(x)N_{1,\varepsilon }^{-}(x)=M_{0,\varepsilon }^{-}(x)+C_{0},$$

where $C_{0}=(c_{ij}^{0})$ is a constant matrix. Hence,

3.33 $$N_{1,\varepsilon }^{+}(x)=\left(\begin{array}{cc} \frac{x+i}{x-i}(m_{0,11}^{+}-c_{11}^{0}) & \frac{x+i}{x-i}(m_{0,12}^{+}-c_{12}^{0})\\ m_{0,21}^{+}-c_{21}^{0} & m_{0,22}^{+}-c_{22}^{0} \end{array}\right)$$

and

3.34 $$N_{1,\varepsilon }^{-}(x)=\left(\begin{array}{cc} m_{0,11}^{-}+c_{11}^{0} & \frac{x-i}{x+i}(m_{0,12}^{-}+c_{12}^{0})\\ m_{0,21}^{-}+c_{21}^{0} & \frac{x-i}{x+i}(m_{0,22}^{-}+c_{22}^{0}) \end{array}\right).$$

For analyticity of $N_{1,\varepsilon }^{+},N_{1,\varepsilon }^{-}$ in the corresponding half-planes, it is necessary and sufficient that the following conditions be fulfilled:

— $c_{11}^{0}=m_{0,11}^{+}(i)$, $c_{22}^{0}=-m_{0,22}^{-}(-i)$;
— the constant $c_{21}^{0}$ is chosen arbitrarily;
— the solvability condition $m_{0,12}^{+}(i)=-m_{0,12}^{-}(-i)$ holds;
— $c_{12}^{0}=m_{0,12}^{+}(i)$.

In the case of the matrix function *N*_*ε*_(*x*) given by (3.27), we have

3.35 $$\begin{array}{rl} N_{1,\varepsilon }^{+}(x) & =(\begin{array}{c} \frac{x+i}{x-i}\left(\frac{-8i(1-e^{-\varepsilon })}{x+i}+\frac{8xi(e^{i\varepsilon x}-e^{-\varepsilon })}{x^{2}+1}-c_{11}^{0}\right)\\ \frac{-6i(1-e^{-\varepsilon })}{x+i}+\frac{4xi(e^{i\varepsilon x}-e^{-\varepsilon })}{x^{2}+1}-c_{21}^{0} \end{array}\\ & \;\times \begin{array}{c} \frac{x+i}{x-i}\left(\frac{12i(1-e^{-\varepsilon })}{x+i}-\frac{12xi(e^{i\varepsilon x}-e^{-\varepsilon })}{x^{2}+1}-c_{12}^{0}\right)\\ \frac{8i(1-e^{-\varepsilon })}{x+i}-\frac{8xi(e^{i\varepsilon x}-e^{-\varepsilon })}{x^{2}+1}-c_{22}^{0} \end{array}) \end{array}$$

and

3.36 $$\begin{array}{rl} & N_{1,\varepsilon }^{-}(x)\\ & \;=\left(\begin{array}{cc} \frac{-8i(1-e^{-\varepsilon })}{x-i}+\frac{8xi(e^{-i\varepsilon x}-e^{-\varepsilon })}{x^{2}+1}+c_{11}^{0} & \frac{x-i}{x+i}\left(\frac{12i(1-e^{-\varepsilon })}{x-i}-\frac{12xi(e^{-i\varepsilon x}-e^{-\varepsilon })}{x^{2}+1}+c_{12}^{0}\right)\\ \frac{-6i(1-e^{-\varepsilon })}{x-i}+\frac{8xi(e^{-i\varepsilon x}-e^{-\varepsilon })}{x^{2}+1}+c_{21}^{0} & \frac{x-i}{x+i}\left(\frac{8i(1-e^{-\varepsilon })}{x-i}-\frac{8xi(e^{-i\varepsilon x}-e^{-\varepsilon })}{x^{2}+1}+c_{22}^{0}\right) \end{array}\right). \end{array}$$

Here

3.37 $$c_{11}^{0}=m_{0,11}^{+}(i)=-4(1-e^{-\varepsilon })-4\varepsilon e^{-\varepsilon }\;\mathrm{and}\;c_{22}^{0}=-m_{0,22}^{-}(-i)=4(1-e^{-\varepsilon })+4\varepsilon \;e^{-\varepsilon },$$

the solvability condition is satisfied

3.38 $$m_{0,12}^{+}(i)=-m_{0,12}^{-}(-i)=6(1-e^{-\varepsilon })+6\varepsilon \;e^{-\varepsilon },$$

and thus the constant $c_{12}^{0}$ can be chosen accordingly

3.39 $$c_{12}^{0}=m_{0,12}^{+}(i)=6(1-e^{-\varepsilon })+6\varepsilon \;e^{-\varepsilon }.$$

Finally, the constant $c_{21}^{0}$ can be chosen arbitrarily.

Thus, the first-order approximation $G_{1,\varepsilon }^{*}(x)$ for the factorization of *G*_*ε*_(*x*) is given by the following formula:

3.40 $$G_{1,\varepsilon }^{*}(x):=(I+N_{1,\varepsilon }^{-}(x)(\Lambda^{+}{\left(x)\right)}^{\left(-1\right)})\Lambda (x)(I+(\Lambda^{-}{\left(x)\right)}^{\left(-1\right)}N_{1,\varepsilon }^{+}(x)),$$

where matrices $N_{1,\varepsilon }^{\pm }(x)$ are presented in (3.35) and (3.36) with the above-described choice of constants.

In order to estimate the quality of the approximation, it is customary to define the following remainder matrix:

3.41 $$\Delta K_{1,\varepsilon }(x):=G_{\varepsilon }(x)-G_{1,\varepsilon }^{*}(x).$$

Direct calculations show that *ΔK*_1,*ε*_(*x*)=*O*(*ε*^2^) as *ε*→+0 and thus $G_{1,\varepsilon }^{*}(x)$ is the 2-guided perturbation for the matrix *G*_*ε*_(*x*).

**Remark 3.8**

Matrix *ΔK*_1,*ε*_(*x*) has an interesting behaviour, as a consequence of a special property of the matrix *N*_*ε*_(*x*): *n*_11_=−*n*_22_. Namely, it tends to the diagonal matrix as x→∞, specifically,

3.42 $$\Delta K_{1,\varepsilon }(\infty )=\left(\begin{array}{cc} {\left(c_{11}^{0}\right)}^{2}+c_{12}^{0}c_{21}^{0} & 0\\ 0 & {\left(c_{22}^{0}\right)}^{2}+c_{12}^{0}c_{21}^{0} \end{array}\right).$$

Thus, by taking $c_{21}^{0}=0$ we have

$$\Delta K_{1,\varepsilon }(\infty )=16{\left(1-e^{-\varepsilon }+\varepsilon \;e^{-\varepsilon }\right)}^{2}I,$$

and by taking $c_{21}^{0}=-\frac{8}{3}(1-e^{-\varepsilon }+\varepsilon \;e^{-\varepsilon })$ we have

$$\Delta K_{1,\varepsilon }(\infty )=\left(\begin{array}{cc} 0 & 0\\ 0 & 0 \end{array}\right).$$

The above two characteristic values of the constant $c_{21}^{0}$ will be used in our numerical description of the behaviour of the remainder *ΔK*_1,*ε*_(*x*) of the first-order approximate factorization of the matrix (3.25).

In this example, we have restricted our calculation to only the first step of the approximation. In principle, the procedure for the next steps has already been described. However, there is no guarantee that the next step will be successful and a higher order guided perturbation will have been derived.

### (e) Example of a matrix which does not satisfy the solvability conditions

Simple changes to the matrix *G*_*ε*_(*x*) can lead to a violation of the solvability conditions for the corresponding boundary value problem. Let us consider

3.43 $${\hat{G}}_{\varepsilon }(x)=\left(\begin{array}{cc} \frac{x^{2}+xi(-18+8\;e^{i\varepsilon x}+8\;e^{-i\varepsilon x})-1}{x^{2}+1} & \frac{xi(24-16\;e^{i\varepsilon x}-8\;e^{-i\varepsilon x})}{x^{2}+1}\\ \frac{xi(-12+4\;e^{i\varepsilon x}+8\;e^{-i\varepsilon x})}{x^{2}+1} & \frac{x^{2}+xi(18-8\;e^{i\varepsilon x}-8\;e^{-i\varepsilon x})-1}{x^{2}+1} \end{array}\right).$$

As before, ${\hat{G}}_{0}(x)=G_{0}(x)$, and thus ${\hat{G}}_{0}(x)$ possesses a factorization with partial indices $\varkappa_{1}=1,\varkappa_{2}=-1$.

We note that

$${\hat{G}}_{\varepsilon }(x)=G_{\varepsilon }(x)+\left(\begin{array}{cc} 0 & -\frac{xi(4\;e^{i\varepsilon x}-4\;e^{-i\varepsilon x})}{x^{2}+1}\\ 0 & 0 \end{array}\right).$$

We apply the above-described asymptotic procedure to our matrix ${\hat{G}}_{\varepsilon }(x)$, and show that this matrix cannot possess a bounded first-order asymptotic factorization with the same partial indices as ${\hat{G}}_{0}(x)$.

The corresponding matrix is given by

3.44 N^ε(x):=G^ε(x)−Λ(x)=(xi(−16+8 eiεx+8 e−iεx)x2+1xi(24−16 eiεx−8 e−iεx)x2+1xi(−12+4 eiεx+8 e−iεx)x2+1xi(16−8 eiεx−8 e−iεx)x2+1)=x sin⁡(εx/2)x2+1(−32i sin⁡εx22i(24 sin⁡εx2+icos⁡εx2)−24i(sin⁡εx2−icos⁡εx2)32i sin⁡εx2).

The first step of the asymptotic factorization leads to the problem

3.45 $$\Lambda^{+}(x){\hat{N}}_{1,\varepsilon }^{+}(x)+{\hat{N}}_{1,\varepsilon }^{-}(x)\Lambda^{-}(x)={\hat{M}}_{0,\varepsilon }(x),$$

where the matrix function ${\hat{M}}_{0,\varepsilon }(x)={\hat{N}}_{\varepsilon }(x)$ can be represented in the following form:

3.46 $${\hat{M}}_{0,\varepsilon }(x)={\hat{M}}_{0,\varepsilon }^{+}(x)+{\hat{M}}_{0,\varepsilon }^{-}(x)$$

and

3.47 $${\hat{M}}_{0,\varepsilon }^{+}(x)=\left(\begin{array}{cc} \frac{-8i(1-e^{-\varepsilon })}{x+i}+\frac{8xi(e^{i\varepsilon x}-e^{-\varepsilon })}{x^{2}+1} & \frac{12i(1-e^{-\varepsilon })}{x+i}+\frac{-16xi(e^{i\varepsilon x}-e^{-\varepsilon })}{x^{2}+1}\\ \frac{-6i(1-e^{-\varepsilon })}{x+i}+\frac{4xi(e^{i\varepsilon x}-e^{-\varepsilon })}{x^{2}+1} & \frac{8i(1-e^{-\varepsilon })}{x+i}+\frac{-8xi(e^{i\varepsilon x}-e^{-\varepsilon })}{x^{2}+1} \end{array}\right)$$

and

3.48 $${\hat{M}}_{0,\varepsilon }^{-}(x)=\left(\begin{array}{cc} \frac{-8i(1-e^{-\varepsilon })}{x+i}+\frac{8xi(e^{-i\varepsilon x}-e^{-\varepsilon })}{x^{2}+1} & \frac{12i(1-e^{-\varepsilon })}{x+i}+\frac{-8xi(e^{-i\varepsilon x}-e^{-\varepsilon })}{x^{2}+1}\\ \frac{-6i(1-e^{-\varepsilon })}{x+i}+\frac{8xi(e^{-i\varepsilon x}-e^{-\varepsilon })}{x^{2}+1} & \frac{8i(1-e^{-\varepsilon })}{x+i}+\frac{-8xi(e^{-i\varepsilon x}-e^{-\varepsilon })}{x^{2}+1} \end{array}\right).$$

Bounded solutions to (3.45) have to satisfy the relation

3.49 $${\hat{N}}_{1,\varepsilon }^{+}(x)=(\Lambda^{+}{\left(x)\right)}^{-1}[{\hat{M}}_{0,\varepsilon }^{+}(x)-{\hat{C}}_{0}],\;{\hat{N}}_{1,\varepsilon }^{-}(x)=[{\hat{M}}_{0,\varepsilon }^{-}(x)+{\hat{C}}_{0}](\Lambda^{-}{\left(x)\right)}^{-1},$$

where ${\hat{C}}_{0}=({\hat{c}}_{i}j^{0})$ is a constant matrix.

In this case,

$${\hat{m}}_{0,12}^{+}(i)=6(1-e^{-\varepsilon })-8\varepsilon \;e^{-\varepsilon }\;\mathrm{and}\;{\hat{m}}_{0,12}^{-}(-i)=-6(1-e^{-\varepsilon })+4\varepsilon \;e^{-\varepsilon },$$

and thus, the solvability condition ${\hat{m}}_{0,12}^{+}(i)=-{\hat{m}}_{0,12}^{-}(-i)$ is satisfied only for *ε*=0. For all *ε*≠0, there is no approximate solution (up to *ε*^2^) of the functional equation (similar to (3.30)).

**Remark 3.9**

Note that, by construction,

$${\hat{G}}_{\varepsilon }(x)-G_{\varepsilon }(x)=\left(\begin{array}{cc} 0 & O(\varepsilon )\\ 0 & 0 \end{array}\right),\;\varepsilon \to 0.$$

Hence, ${\hat{G}}_{\varepsilon }(x)$ presents an example of the regular perturbation of ${\hat{G}}_{0}(x)$, for which no regular *k*-guided perturbation (*k*>1) exists while construction of a singular perturbation remains an open problem.

## 4. Numerical examples and discussion

In this section, we analyse the quality of the approximation provided by the 2-guided perturbation performed in §3d.

First, we consider the case when *c*_21_=0, and thus the limiting value of the remainder, *ΔK*_1*ε*_ does not vanish at infinity. Specifically, we estimate the element on the main diagonal in the following way:

$$\Delta k_{jj}(\infty )=16(1-e^{-\varepsilon }+\varepsilon \;e^{-\varepsilon })=64\varepsilon^{2}-96\varepsilon^{3}+O(\varepsilon^{4}),\;\varepsilon \to 0,\;j=1,2.$$

**Figure 2.**
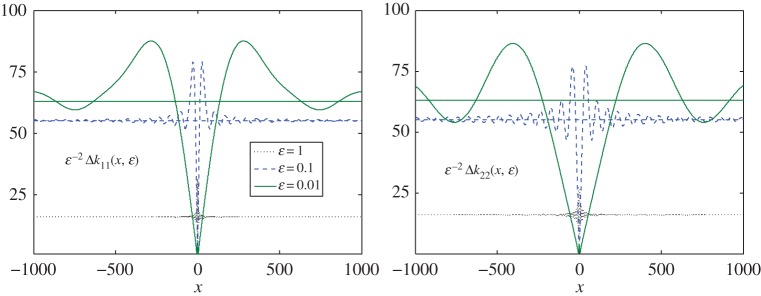
Diagonal elements, *Δk*_*jj*_(*x*,*ε*), *j*=1,2, of matrix *ΔK*_1,*ε*_(*x*) defined for various values of parameter *ε*, the constant $c_{21}^{0}=0$. The elements are normalized to the value of parameter *ε*^2^. (Online version in colour.)

In figure 2*a*,*b*, those components are presented in their normalized forms. We can see that the estimate is true (see the discussion on the small parameter following formula (3.41)). Furthermore, the matrix converges to its limiting values more quickly for larger values of the small parameter, while the oscillations decay more slowly for smaller values.

**Figure 3.**
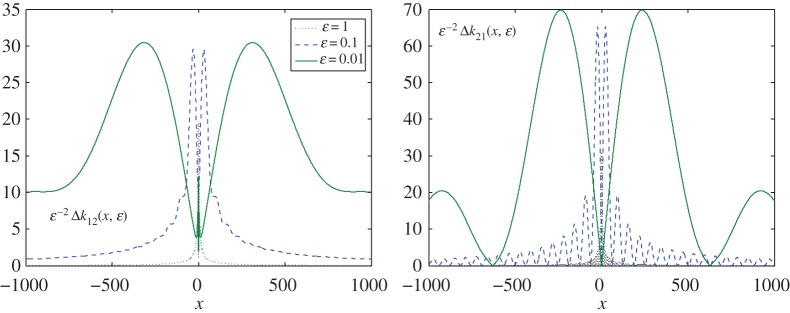
The other two elements, *Δk*_*ij*_(*x*,*ε*), *i*+*j*=3, of matrix *ΔK*_1,*ε*_(*x*) for *ε*=1;0.1;0.01, and constant $c_{21}^{0}=0$. It is clear that both entries vanish, i.e. *k*_*ij*_→0 as |x|→∞. (Online version in colour.)

In figure 3*a*,*b*, the remaining two components are depicted in the same normalized forms. Preserving the same estimate, where *ΔK*_1,*ε*_(*x*)=*O*(*ε*^2^) as *ε*→0, the components now decay to *O*(*x*^−1^), as |x|→∞. The trend is also clearly visible here, that the smaller *ε* is, the slower it converges to its limiting value. In other words, the small parameter *ε* determines the magnitude of the reminder matrix, but the oscillations are larger in this case, and more pronounced along the real axis.

Interestingly, the components on the main diagonal are comparable in value, but not equal, while the remaining two differ in value by almost a factor of two. Moreover, the latter are also two times smaller in magnitude than the diagonal elements.

**Figure 4.**
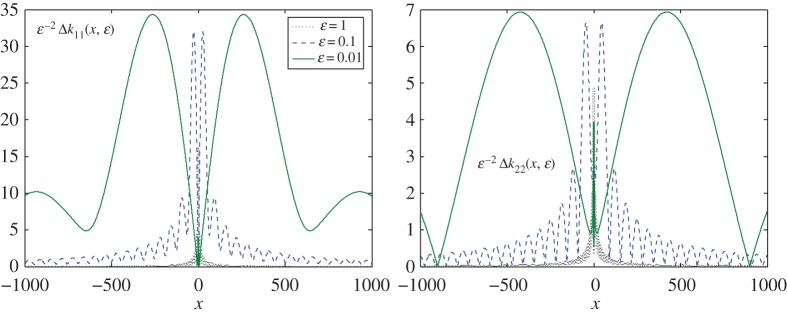
Diagonal elements, *Δk*_*jj*_(*x*,*ε*), *j*=1,2, of matrix *ΔK*_1,*ε*_ for various *ε* and the constant $c_{21}^{0}=-\frac{8}{3}(1-e^{-\varepsilon }+\varepsilon \;e^{-\varepsilon })$. The elements are normalized to the value of parameter *ε*^2^. The horizontal lines show the limiting values of the normalized components at infinity. (Online version in colour.)

**Figure 5.**
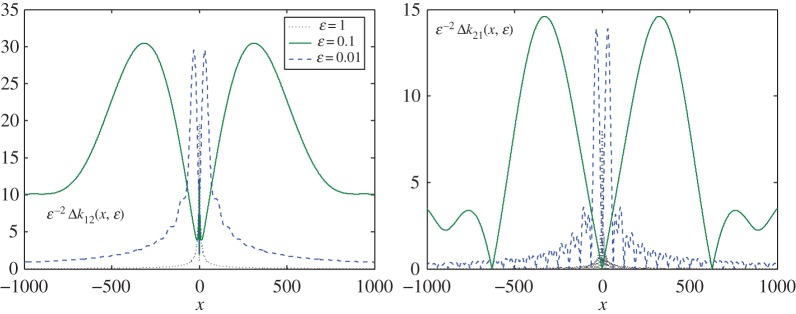
The other two elements *Δk*_*ij*_(*x*,*ε*), *i*+*j*=3, of matrix *ΔK*_1,*ε*_(*x*) for *ε*= 1;0.1;0.01, and the constant $c_{21}^{0}=-\frac{8}{3}(1-e^{-\varepsilon }+\varepsilon \;e^{-\varepsilon })$. It is clear that both entries vanish, i.e. *k*_*ij*_→0 as |x|→∞. (Online version in colour.)

The situation changes when we consider the second case, where $c_{21}^{0}=-\frac{8}{3}(1-e^{-\varepsilon }+\varepsilon \;e^{-\varepsilon })$. The respective graphs are presented in figures 4 and 5. Now, all the components decay at infinity as *O*(*x*^−1^), as |x|→∞, and simultaneously have the same estimate of (*O*(*ε*^2^)) when *ε*→0, as predicted. The magnitudes of the components are, however, more balanced in the sup norm $∥\Delta K_{1,\varepsilon }^{\left(1\right)}∥>2∥\Delta K_{1,\varepsilon }^{\left(2\right)}∥$. This demonstrates that we can choose an optimal approximation preserving some specified requirement by varying the value of the arbitrary constant *c*_21_. Comparing these two cases, it is clear that the second is preferable to the first for the reasons discussed earlier.

Any specific factorization will of course require its own analyses. However, if the estimate

$$G_{\varepsilon }(x)=o(1),\;|x|\to \infty ,$$

is true, then the reminder can be estimated by

$$\Delta K_{1,\varepsilon }(x)={\left(\begin{array}{cc} c_{11}^{0} & c_{12}^{0}\\ c_{21}^{0} & c_{22}^{0} \end{array}\right)}^{2}+o(1),\;|x|\to \infty .$$

We note that this property may change in the next step if we wish to and can continue the approximation procedure (the conditions will remain valid for the next step). Here, the limiting values for the first step will also play their role. We can deliver a similar formula based on the two consequent approximations, where two sets of constants will then be involved: *c*_*jl*_ (first step) and *d*_*jl*_ (second step), *j*,*l*=1,2.

Judging by the magnitude of the reminder for both the presented examples, we can conclude that the 2-guided perturbation may be sufficient for practical purposes. Thus, if even one approximation step is practically possible, meaning that conditions (3.12)–(3.16) are satisfied, then we can use this approximation directly in solving the Wiener–Hopf equation.

To close, we must highlight that, if conditions (3.12)–(3.16) for matrix *G*_*ε*_, with unstable partial indices, are not satisfied, the question of how to compute a valuable approximate factorization for such a matrix-function remains open.

## Data accessibility

This paper has no additional data.
